# Mechanotransductive Receptor Piezo1 as a Promising Target in the Treatment of Neurological Diseases

**DOI:** 10.2174/1570159X20666220927103454

**Published:** 2023-08-15

**Authors:** Natalia Bryniarska-Kubiak, Andrzej Kubiak, Agnieszka Basta-Kaim

**Affiliations:** 1 Laboratory of Immunoendocrinology, Department of Experimental Neuroendocrinology, Maj Institute of Pharmacology, Polish Academy of Sciences, 12 Smętna St., Kraków, 31-343, Poland;; 2 Laboratory of Stem Cell Biology, Faculty of Biochemistry, Biophysics and Biotechnology, Jagiellonian University, Kraków, Poland

**Keywords:** Piezo1 receptor, Alzheimer's disease, ischemic stroke, GsMTx4, mechanotransduction, biomechanics

## Abstract

In recent years, increasing attention has been paid to the role of physical factors in biological processes. This direction was ultimately confirmed by the recent 2021 Nobel Prize in medicine and physiology awarded in ½ to Ardem Patapoutian for his discovery of Piezo1 and Piezo2 mechanosensitive receptors. Among them, Piezo2 is responsible for sensing touch, while Piezo1 is engaged in a variety of mechanotransduction events. Piezo1 is expressed in various central nervous system cells, while its expression may be affected in the course of various pathological conditions. Recently, thanks to the development of Piezo1 modulators (*i.e*. Yoda1, Jedi1/2 and Dooku2), it is possible to study the role of Piezo1 in the pathogenesis of various neurological diseases including ischemia, glioma, and age-related dementias. The results obtained in this field suggest that proper modulation of Piezo1 receptor might be beneficial in the course of various neurological diseases.

## 1 INTRODUCTION

In recent years increasing attention has been paid to the role of physical interactions in various biological processes including development [1], stem cell niche interactions [2, 3], cancer [4, 5], infections [6], inflammation [7, 8] and central nervous system functioning [9, 10]. The importance of this direction of research was ultimately confirmed by the recent Nobel Prize in medicine and physiology awarded to David Julius for his work on TRPV1 and TRPM8 ion channels activated by heat pain as well as to Ardem Patapoutian for his research on mechanosensitive receptors Piezo1 and Piezo2 [11]. In the pioneering studies, they showed an important role of Piezo2 in touch perception and originally identified the functionality of Piezo1 receptor using mechanosensitive neuroblastoma cell line, namely neuro2A [12]. In the following years, the role of Piezo1 was shown in several tissues and processes including neurological diseases [13] and inflammation [14]. Correspondingly, it is known that multiple neurological diseases are still lacking successful treatment (*i.e*. stroke, Alzheimer's disease, Parkinson's disease, Huntington's disease, and others). Thus, in the present work, we aim to comment on recent developments in studies on the role of Piezo1 in neurological disease-associated processes and consequently, to assess the possibility of targeting this protein in the therapy of various neurological diseases.

## 2 PIEZO1 MECHANISM OF ACTION

The functional Piezo1 receptor is a trimer built of three identical proteins. Each is 2547 amino acids in length and is organized into a domain building central pore (about 360 amino acids on C-terminus), linker, beam, and blade-like structures – considered as a mechanosensitive part of the receptor [15-17]. The proposed mechanisms of its action are allosteric. Three blade-like domains are in direct contact with the cell membrane and stress applied to the membrane leads to changes in their spatial location thus leading to the opening of the ion channel [18, 19]. In the recent pioneering work, Yang *et al.* examined the mechanism of action of Piezo1 embedded in liposome membrane which allows for investigation of Piezo1 structure in more physiological conditions. They were able to observe the deformation of the whole system of Piezo1-lipid bilayer providing a better explanation of the gating mechanism and in turn calculating a half-maximal activation tension for this channel – 1.9 pN/nm [20]. Importantly, apart from activation by a physical trigger, Piezo1 can be activated by small-molecule agonist Yoda1 (Table **1**). Yoda1 possesses its binding pocket between Repeat A and N-terminal parts of an implicit mechano-sensing blade-like domain. This binding was proposed to work on principle on the wedge localized between the aforementioned domains. It results in the extension of the blade-like domain which leads to ion channel opening in conditions when stress applied to the membrane is not sufficient to open the channel alone [21]. Importantly, not only Yoda1 was reported as a Piezo1 activity modulator. Jedi1 and Jedi2 which are structurally different from Yoda1 are other small molecules that pharmacologically trigger Piezo1 activation (Table **1**) [15]. The landscape of pharmacological modulation of the Piezo1 receptor was also expanded with the development of novel Yoda1 analogs. By chemical modification of the pyrazine ring of the Yoda1 compound, Evans *et al.* [22] developed a compound capable of antagonizing the Yoda1 action on Piezo1. Sticking to the naming convention of Piezo1 modulators (which refers to the Star Wars saga) they named this compound Dooku1 (Table **1**). The use of Dooku1 led to inhibition of relaxation of aortic rings induced by Yoda1 action on Piezo1 [22]. What is more, the GsMTx4 peptide was shown to inhibit Piezo1 activity in HEK293 cells transfected with Piezo1 (Table **1**) [23, 24].

Interestingly, Piezo1 function could be affected by dietary fatty acids. In principle supplementation of NA2 cells with margaric acid (member of saturated fatty acids) was inhibiting mechanically inducted activation of Piezo1 in a concentration-dependent manner. This inhibition resulted from the stiffening of the cell membrane and was reversible after the use of Piezo1 agonist – Yoda1 proving that margaric acid was affecting the functionality of Piezo1 but not its expression [25].

Therefore, the accessibility of modulators and the important role of Piezo1 in multiple biological processes make it an attractive potential therapeutic target.

## 3 PIEZO1 IN THE NERVOUS SYSTEM

**Fig. (1):**
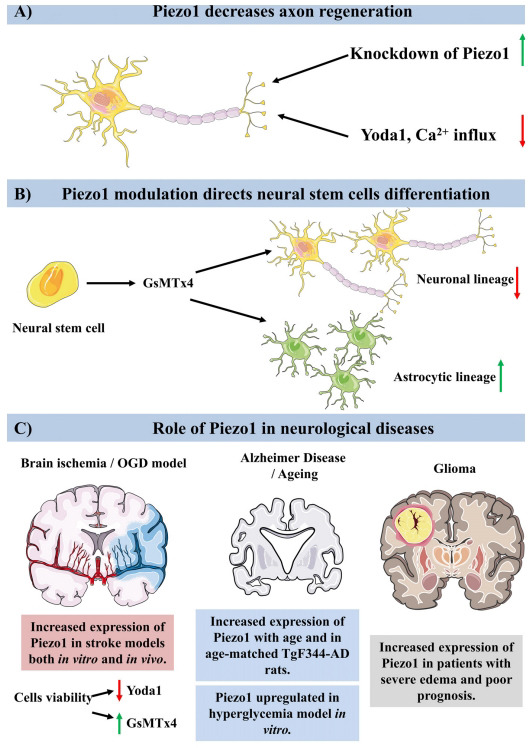
Role of Piezo1 in nervous system functioning and pathologies. (**A**) Piezo1 decreases axon regeneration. Knockdown of Piezo1 homolog in *Drosophila melanogaster* enhance axon regeneration. Consequently, both Piezo1 homolog dependent Ca^2+^ current were associated with inhibition of axon growth in *Drosophila melanogaster* as well as the use of Yoda1 – Piezo1 agonist to inhibit axon regeneration in mammalian cell culture [29]. (**B**) Piezo1 modulation directs human neural stem cell differentiation *in vitro*. Piezo1 antagonist – GsMTx4 inhibit differentiation of neural stem cells into neruogenic lineage while favoring their differentiation into the astrocytic lineage [26]. (**C**) Role of Piezo1 in neurological diseases. Brain ischemia: Expression of Piezo1 was increased in stroke models both *in vitro* as well as *in vivo*. Use of Piezo1 agonist Yoda1 led to increased cell death, while the use of Piezo1 antagonist GsMTx4 led to decreased cell death after oxygen glucose deprivation on PC12 cells [32]. Alzheimer's Disease and aging: Both aged and Alzheimer's disease model rats exhibit increased expression of Piezo1. Piezo1 was upregulated in *in vitro* model of hyperglycemia [33, 34]. Glioma: Expression of Piezo1 was elevated in patients with edema and poor prognosis [30, 31].

As mentioned above, physical factors play a crucial role both in the functioning of stem cells and in developmental processes. Importantly, the activation of Piezo by a physical trigger leads to an influx of Ca^2+^ ions inside the cell which contributes to the differentiation of neural stem cells. Pathak *et al.* showed that the use of the Piezo1 inhibitor GsMTx4 led to inhibition of differentiation of human neural stem cells into the neuronal lineage and promoted their differentiation into astrocytic lineage (Fig. **1B**) [26]. Nevertheless, it should be pointed out that the authors of this work performed their experiments using only *in vitro* model of primary human neural stem cells. In the context of stem cells, research conducted *in vivo* is crucial to confirm their physiological functionality, especially due to multiple overinterpretations which had arisen based on only *in vitro* differentiation experiments [27, 28]. Interestingly, Song *et al.* showed that the mammalian Piezo1 homolog-dependent Ca^2+^ current was associated with inhibition of axon growth in *Drosophila melanogaster*. Consequently, they observed that the knockdown of this receptor increased axon regeneration. Corresponding results were obtained by the authors of this research also in the mammalian system, where the use of Piezo1 agonist Yoda1 led to decreased axon regeneration of rat hippocampal neurons cultured in the microfluidic chamber (Fig. **1A**) [29].

The importance of Piezo1 was also observed in the context of brain tumors. Analysis of transcriptomic data from the following databases: Chinese Glioma Genome Atlas RNAseq, the Cancer Genome Atlas RNAseq, and the GSE16011 database, revealed that Piezo1 expression was shown to be correlated with the clinical and molecular characteristics of glioma. What is more, elevated expression of Piezo1 was associated with poor prognosis (Fig. **1C**) [30-34]. Corresponding results were also obtained by Qu *et al.* who observed increased expression of Piezo1 in human tumor tissue. Furthermore, Piezo1 expression was elevated in patients with severe edema (Fig. **1C**) [30-34]. Importantly authors investigated Piezo1 expression (both mRNA and protein) in commercially available cell lines. They showed that the level of Piezo1 expression in glioma cell lines (U251, LN319, SNB19) was higher compared to the astrocytic control cell line (HA1800). On the other hand, although the level of Piezo1 did not differ significantly from control cells in the case of other glioma cell lines (S683, SW1783, U373) investigated in this study. Such observations strongly indicate that the possible treatment of Piezo1 as a brain tumor marker must be considered very carefully [31].

Recently, the role of Piezo1 in the mechanisms of age-related physiological and pathological dementias, like in the course of Alzheimer’s disease (AD) and of other origins, is postulated. Glia are highly mechanosensitive cells that can recognize abnormal stiffening of brain tissue caused by amyloid-beta (Ab) aggregates – AD hallmarks. In principle, Ab stiffness might be as high as ~3 × 10^6^ kPa while brain tissue is one of the softest within the body – with elastic modules around 0.5 kPa. Velasco-Estevez *et al.* (2018), found that ageing results in an increase in Piezo1 protein level in the cortex and hippocampus of rats. Moreover, its level is significantly increased in age-matched TgF344-AD rats (Fig. **1C**). TgF344-AD rats might be used as a model animal for AD due to overexpression of human Amyloid precursor protein (APP) with the Swedish mutation and mutated Presenilin-1 (PSEN1). Importantly, the comorbidity of peripheral infection (urinary tract) caused a further elevation of Piezo1 gene expression suggesting the impact of pro-inflammatory mediators released by macrophages/microglia in regulating astrocytic Piezo1 levels. Finally, they showed that Piezo1 regulates the physical communication between neurons and astrocytes because its blockade with GsMTx4 promotes the decoupling of neurons from astrocytes and leads to fewer cell-cell contacts [33].

Interestingly, Ab is considered not only a marker of Alzheimer’s Disease but its elevated concentration both in brain tissue as well as in cerebral spinal fluid is observed in a course of traumatic brain injury (TBI). In this context, impact of Ab on the mechanosensitivity of cells mediated by Piezo1 was examined *in vitro*. For this purpose authors use HEK293 cells with integrated cDNA for fluorescently tagged Piezo1 protein. To probe Piezo1 activity, cells grown in a fluidic chamber were treated with a Fluo-4 AM calcium sensor and treated with a shear stress of 15 dynes/cm^2^. Such conditions allowed us to measure enhanced calcium influx in Piezo1 labeled cells in response to shear stress. Consequently, the use of Ab monomers significantly decreased the shear stress activated response of cells. Although while Ab was present in form of oligomers its inhibitory properties were significantly lower. What is more, authors showed that Ab might decrease motility of cell in a very robust way (10-fold) in wound-healing assay. Although, what was also underlined by the authors, one potential limitation of this study is the fact that observation of Piezo1 related events takes place using cells with Piezo1 overexpression what might introduce bias in physiological role of this protein in processes cells motility [35].

On the other hand, Ivkovic *et al.* (2022) found that although Piezo1 expression was increased in cultured primary astrocytes stimulated with bacterial endotoxin, lipopolysaccharide (LPS), the production of pro-inflammatory cytokines (*e.g*. IL-1β, TNF-α, IL-6) was unaffected following GsMTx4 administration, while stimulation with Yoda1 diminished the release of the mentioned proinflammatory factors [36]. Consequently, more studies are necessary to determine the role of Piezo1 in pathological conditions related to glia functioning.

While considering the role of Pizeo1 in various dementias, it should also be stressed that there is a potential interaction of Piezo1 with the metabolic status of cells, and its changes may be crucial in cognitive declines. Liu *et al.* (2021) evaluated the role of Piezo1 in microglial dysfunction in acute hyperglycemia in an *in vitro* microglia model using BV2 microglia cell line. The authors revealed that Piezo1 was upregulated (Fig. **1C**). Simultaneously, an increase in the cytosolic Ca^2+^ content in a high glucose environment, and impaired reactivity to inflammatory activation (Ab or LPS) were associated with Piezo1 activation, while inhibition of Piezo1 could rescue microglial dysfunction. Moreover, the authors found that the molecular mechanism of Piezo1 downstream mechanisms involves JNK1 and mTOR pathways. This new observation for the first time underlined the involvement of Piezo1 and its downstream signaling pathways in diabetic cognitive dementias [34]. Nevertheless, since this research was conducted on an immortalized cell line derived from C57/BL6 mouse, it will be beneficial to address this topic in an animal model or at least at the level of co-culture or tissue culture, to investigate complex and physiological cell-cell interactions, which are extremely crucial in the context of neuroscience.

Consequently, the study of small-molecule drugs able to inhibit Piezo1 may pave the way for a potential pharmacological intervention to treat neurocognitive deficits or other neurological diseases associated with Piezo1 overexpression. While considering pharmacological modulation of Piezo1 action, caution should be kept due to the fact that Piezo1 plays a significant role in functioning endothelial cells, as its modulation resulting from system delivery of modulators might affect the functioning of the cardiovascular system.

## 4 PIEZO 1 IN NEUROVASCULAR DISEASES

As mentioned above Piezo1 is intensively investigated in the context of endothelial cells. In principle, they act as sensors of blood flow-induced shear stress in blood vessels. They might be activated by force acting on the cell membrane of endothelial cells resulting in Ca^2+^ entry into cells [37]. The significance of Piezo1 in the context of the cerebrovascular system has been recently investigated. Murine endothelial cells isolated from both cortical and retinal capillaries expressed functional Piezo1 receptors. What is more, *ex vivo* investigations on cerebral blood flow in the retina indicated that both pharmacological (Ruthenium Red) and genetic manipulation of Piezo1 in endothelial cells decreased significantly the ability of those cells to respond to mechanical stimuli [38]. Consequently, the role of Piezo1 was lately indicted in the pathogenesis of cerebral cavernous malformation which is an inherited disease of brain capillaries. Under this pathological condition, junctions between capillary building cells are malformed which leads to the permeability of the blood-brain barrier. Whole exome sequencing provided evidence for the existence of a missense mutation in the Piezo1 locus which indicated that Piezo1 dysfunction might contribute to the development of cerebral cavernous distortion [39]. While considering the role of Piezo1 in brain diseases associated with brain vasculature, it should also be emphasized that its significance was examined in the context of brain ischemia. Wang *et al.* investigated the role of Piezo1 in reperfusion injury in the oxygen-glucose deprivation (OGD) stroke model on PC12 cells and also *in vitro* using the middle cerebral artery occlusion (MCAo) model in rats. The authors noticed increased expression of Piezo1 in both PC12 cells undergoing OGD and in the cortex of rats after MCAo and reperfusion (Fig. **1C**). Importantly, the activation of Piezo1 by Yoda1 led to increased cell death in response to OGD while the use of the Piezo1 inhibitor GsMTx4 peptide resulted in decreased cell death after OGD (Fig. **1C**) [32].

## CONCLUSION

In recent years, the number of publications in Scopus about Piezo1 has gradually been increasing starting from 2 per year in 2010 to 195 per year in 2021. This trend is ultimately underlain by the significant role of this mechanosensitive receptor in a variety of biological processes. On the other hand, Piezo1/2 boom should lead researchers to prudence about results presented in new scientific publications – in the context of also physiological meaning, data reproducibility and their scientific merit [40, 41]. Recent years provide increasing evidence that physical processes might play a very important role in nervous system development, functioning, and diseases [42]. Stress generated by solid brain tumors was even shown to be a factor strong enough to lead to neuronal nuclei deformation followed by their apoptosis but also to occlusion of blood vessels in proximity to the solid tumor [43]. At the same time, it should be underlined that proper adjustment of nanotopography of substrate for PC12-neuroblast cell culture increased their differentiation by induction of mechanotransduction signaling [44]. Given this, the role of mechanotransductive events is very important. As presented in this work, Piezo1 has been associated with negative outcomes in the case of axon regeneration [29], ischemia [32], and glioma [30, 31]. Consequently, in the aforementioned cases modulation of the Piezo1 receptor resulted in beneficial effects strongly indicating that Piezo1 might be considered a promising target for future therapies for brain diseases. Nevertheless as pointed out in a few cases in this paper, a lot of reliable and precise work on this topic is still required to answer the question whether and possibly how Piezo1 functioning might be translated to clinical application. In this context, not only the development of both proper animal models of particular diseases is extremely important but also advanced 3D cell culture systems for maintaining more complex cell-cell interaction in the context of human cells which could not be achieved in animals.

## Figures and Tables

**Table 1:** Modulators of Piezo1 receptor.

**Name**	**Function**	**References**
Yoda1	Agonist of Piezo1. It binds between Repeat A and N-terminal parts of an mechano-sensing blade-like domain on a principle of the wedge. It results in extension of the blade-like domain which leads to ion channel opening.	[21]
Jedi1, Jedi2	Agonists of Piezo1. It activates Piezo1 through interaction with extracellular regions of the peripheral blade-like domain.	[15]
Dooku1	Generated by chemical modification of Yoda1 pyrazine ring. It does not exhibit agonist activity but it is antagonizing Yoda1 action on Piezo1.	[22]
GsMTx4	4 kDa peptide isolated from tarantula venom. Principle of its action relies on the modification of channel gating. It increases the energy of open state *versus* decreasing energy of the closed state of the receptor.	[23, 25]

## LIST OF ABBREVIATIONS

AD: Alzheimer’s Disease
APP: Amyloid Precursor Protein
TBI: Traumatic Brain Injury
OGD: Oxygen-glucose Deprivation
